# Mapping Soil Properties in the Haihun River Sub-Watershed, Yangtze River Basin, China, by Integrating Machine Learning and Variable Selection

**DOI:** 10.3390/s24123784

**Published:** 2024-06-11

**Authors:** Jun Huang, Jia Liu, Yingcong Ye, Yameng Jiang, Yuying Lai, Xianbing Qin, Lin Zhang, Yefeng Jiang

**Affiliations:** 1 Basic Geological Survey Institute of Jiangxi Geological Survey and Exploration Institute (Jiangxi Nonferrous Geological Mineral Exploration and Development Institute), Nanchang 330045, China; junshi_2005.11@163.com (J.H.); ye_changfeng@163.com (Y.L.); nieyum@163.com (X.Q.); zhanglin361988@sina.cn (L.Z.); 2 College of Land Resources and Environment, Jiangxi Agricultural University, Nanchang 330045, China; liuja1216@163.com (J.L.); yycdayu@126.com (Y.Y.); jiang2070690798@163.com (Y.J.)

**Keywords:** soil properties, recursive feature elimination, random forest, extreme gradient boosting, spatial distribution

## Abstract

Mapping soil properties in sub-watersheds is critical for agricultural productivity, land management, and ecological security. Machine learning has been widely applied to digital soil mapping due to a rapidly increasing number of environmental covariates. However, the inclusion of many environmental covariates in machine learning models leads to the problem of multicollinearity, with poorly understood consequences for prediction performance. Here, we explored the effects of variable selection on the prediction performance of two machine learning models for multiple soil properties in the Haihun River sub-watershed, Jiangxi Province, China. Surface soils (0–20 cm) were collected from a total of 180 sample points in 2022. The optimal covariates were selected from 40 environmental covariates using a recursive feature elimination algorithm. Compared to all-variable models, the random forest (RF) and extreme gradient boosting (XGBoost) models with variable selection improved in prediction accuracy. The *R*^2^ values of the RF and XGBoost models increased by 0.34 and 0.47 for the soil organic carbon, by 0.67 and 0.62 for the total phosphorus, and by 0.43 and 0.62 for the available phosphorus, respectively. The models with variable selection presented reduced global uncertainty, and the overall uncertainty of the RF model was lower than that of the XGBoost model. The soil properties showed high spatial heterogeneity based on the models with variable selection. Remote sensing covariates (particularly principal component 2) were the major factors controlling the distribution of the soil organic carbon. Human activity covariates (mainly land use) and organism covariates (mainly potential evapotranspiration) played a predominant role in driving the distribution of the soil total and soil available phosphorus, respectively. This study indicates the importance of variable selection for predicting multiple soil properties and mapping their spatial distribution in sub-watersheds.

## 1. Introduction

Soil is a basic non-renewable resource that plays an essential role in promoting sustainable agricultural development, maintaining biosphere stability, and tackling climate change [1]. Environmental degradation and food security are prominent problems in agricultural development, which calls for the effective management and protection of soil ecosystems [2]. A watershed is an area of land that drains water into a specific water body. The watershed is considered fundamental to ecological balance, agricultural production, and climate change [3]. Soil properties, such as organic carbon (SOC), total phosphorus (STP), and available phosphorus (SAP) contents, have a major influence on soil fertility, carbon cycling, and environmental quality [4,5]. Investigating the distribution of multiple soil properties in watersheds as hydrologic units can provide insights into the latest status of soil health, allowing for the effective evaluation of agricultural production capacity.

Traditional soil mapping mainly involves manual delineation based on topographic maps and aerial images, which is labor-intensive and has a limited accuracy [6]. The rapid development of 3S technology and access to open environmental data (e.g., relief, remote sensing, and climate) have enabled a growing number of digital soil mapping studies to accurately predict the distribution patterns of soil properties on regional scales [7]. Compared to traditional methods, digital soil mapping techniques produce more objective and replicable results with fewer samples [8]. Machine learning models, exemplified by the random forest (RF) [9] and extreme gradient boosting (XGBoost) models [10], are widely used to predict soil properties.

The RF model has been shown to be an effective prediction method, as it prevents over-learning and provides stable predictions [11,12]. For example, Poggio et al. [13] mapped a set of soil properties (e.g., bulk density, SOC, total nitrogen, and cation exchange capacity) at six soil depths using a quartile RF model with 400 environmental covariates. Safanelli et al. [14] leveraged the RF model with Earth observation data and environmental auxiliary information to map the SOC content and storage, pH, and cation exchange capacity in the topsoil of agricultural land in Brazil. The successful application of XGBoost in soil mapping has also been documented, given its fast computation and excellent performance [15,16]. Huang et al. [17] used 10 different methods, including XGBoost, to predict the distribution of available cadmium in soils. Among the traditional and integrated models used, the XGBoost model yielded the best prediction performance, which was superior to the linear regression model in terms of accuracy. The previous studies have demonstrated the applicability of machine learning models represented by RF and XGBoost for predicting soil properties. These models provide promising tools for the accurate mapping of the soil properties in watersheds.

During digital soil mapping, the number of environmental covariates increases rapidly [18]. Therefore, variable selection is commonly used before fitting the final prediction model. This process can enable rapid model calibration, reduce model complexity, improve prediction performance, avoid multicollinearity, and facilitate map generation [19]. The recursive feature elimination (RFE) algorithm is one of the most popular variable selection methods [13,20,21,22,23,24]. When using the RF model to assess variable importance and to predict the SOC content, He et al. [25] selected the most accurate variable combination for each set of environmental covariates based on RFE. Furthermore, Luo et al. [26] assessed the prediction accuracy of images for soil organic matter across different time periods using the RF model. They determined the performance of RF models with two different variable selection methods, and the model with RFE achieved a higher accuracy. While many studies apply variable selection to obtain the optimal variable combinations, little effort has been devoted to quantifying or visualizing the optimization of model performance after variable selection. Moreover, there is a paucity of studies adopting various modeling approaches to predict multiple soil properties.

Haihun River is a tributary of Xiushui River in the Poyang Lake watershed, Yangtze River basin, China. The Haihun River sub-watershed is an important agricultural area in Jiangxi Province, with typical agro-ecosystems and agro-cultural heritage. Maintaining soil health in the sub-watershed is essential for regional agricultural production and ecological protection. In this study, the hypothesis proposed is that the integration of machine learning techniques and meticulous variable selection may significantly enhance the accuracy of soil mapping. We adopted two common models, RF and XGBoost, to predict the SOC, STP, and SAP in the Haihun River sub-watershed. The performance of the models with and without variable selection was compared to demonstrate the optimization of the prediction accuracy for different soil properties. The spatial distributions of the SOC, STP, and SAP in the study area were mapped using the optimal model with the combination of environmental covariates having the highest predictive ability. The results of this study could be useful for assessing soil health and formulating precise agricultural policies at the sub-watershed scale.

## 2. Materials and Methods

### 2.1. Study Area

**Figure 1:**
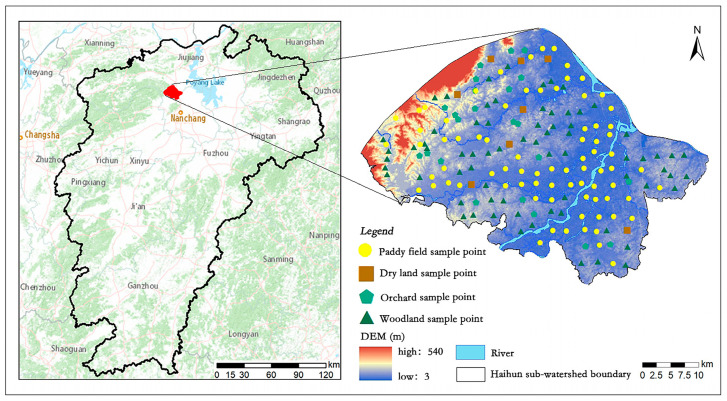
Location of soil sampling points in the study area.

The Haihun River sub-watershed (28°53′–29°7′ N, 115°31′–115°52′ E) is located in the southeastern part of Yongxiu County, Jiujiang City, Jiangxi Province, with a total area of 46,651.11 ha (Figure 1). It is a tributary of Xiushui River and south of the main stream. The sub-watershed is near Anyi County to the south and neighboring Xinjian County to the east. Yongxiu County is situated in the transition zone between the central subtropics and northern subtropics of China. This area experiences a humid monsoon climate characterized by sufficient light and four distinct seasons. There are abundant heat and rainfall, with an annual mean temperature of 16.9 °C and an annual mean rainfall of 1485.30 mm. The major type of land use is woodland followed by cropland. Based on the soil survey results of the Second National Soil Census, a total of 22 soil types are distributed in Yongxiu County, with paddy soil (49.53%) and red soil (20.27%) being predominant

### 2.2. Soil Sampling and Laboratory Analysis

Field sampling was conducted in October 2022 taking into account the uniformity of spatial distribution and the representativeness of sample points. We selected plots in 1.5 km × 1.5 km grids and collected soil samples from a depth of 0–20 cm at five points per plot. The samples from the same plot were thoroughly mixed by the quartering method, and 1 kg samples were retained for the analysis of the soil properties. All samples were air-dried upon arrival in the laboratory. After removing large roots and stones, the samples were ground and sieved (2 mm). To determine the SOC, the samples were heated in an oil bath with potassium dichromate (K_2_Cr_2_O_7_). The STP and SAP were determined by the molybdenum antimony antimony colorimetric method after an alkali fusion of the samples with NaOH and extraction with NaHCO_3_, respectively [27].

The longitudinal and latitudinal positions of each sample point were recorded using a portable global positioning system (GPS) (Garmin Ltd., Olathe, KS, USA). The land use type and soil type around the sample points were also recorded in detail. A total of 180 samples were collected in this study (Figure 1). The outliers were detected by three standard deviations, and the data after removing the outliers were used in this study.

### 2.3. Selection of Environmental Covariates

Zhu et al. [28] supposed that the more similar geographic configurations of two areas, the more similar the values of the target variable at these two areas. Therefore, we assume that the more similar the environmental configurations in which the soils are embedded, the more similar the soil properties will be. Topsoil properties are controlled by the interactions of the initial soil conditions, natural environmental factors, and human activities [29]. Here, we used 40 environmental factors of six types as the initial covariates (Table 1). The optimal covariates were selected as environmental predictors by variable selection.

#### 2.3.1. Soil Covariates

The spatial distribution data of the soil texture were obtained from the National Earth System Science Data Center https://www.geodata.cn/ (accessed on 5 November 2023) and classified into three categories: sand content (Sand), silt content (Silt), and clay content (Clay). The soil bulk density (BD) and pH were derived by interpolating data from the collected soil samples. The soil moisture (SM) data were retrieved from the National Tibetan Plateau Science Data Center https://data.tpdc.ac.cn/ (accessed on 12 January 2024). The bare soil index (BSI) was extracted from Landsat 8 remote sensing images. The soil erosion (SE) data were sourced from a previous publication [30].

#### 2.3.2. Climate Covariates

The annual mean wind speed (WIN), annual mean ground surface temperature (GST), and annual mean relative humidity (RHU) data were obtained from the Resource and Environmental Sciences Data Center of the Chinese Academy of Sciences https://www.resdc.cn/ (accessed on 1 November 2023). The annual maximum temperature (Tmax), annual minimum temperature (Tmin), annual mean temperature (Tmean), and annual mean precipitation (PRE) data were obtained by the revision and downscaling of ERA5-LAND [31].

#### 2.3.3. Remote Sensing Covariates

Landsat 8 remote sensing imagery with a 30 m resolution (11 September 2019) was downloaded from the Geospatial Data Cloud platform https://www.gscloud.cn/ (accessed on 11 October 2023). The first three principal components, PCA1, PCA2, and PCA3, were obtained after radiometric calibration and atmospheric correction implemented using ENVI 5.3 (Harris Geospatial Solutions Inc., Broomfield, CO, USA).

#### 2.3.4. Organism Covariates

The normalized difference vegetation index (NDVI) data came from the National Ecological Data Center https://www.nesdc.org.cn/ (accessed on 19 July 2023), and the dataset was obtained by processing Landsat 5/7/8 remote sensing data based on the Google Earth Engine cloud computing platform. The vegetation net primary productivity (NPP), net ecosystem productivity (NEP), and gross primary productivity (GPP) data were collected from the National Earth System Science Data Center https://www.geodata.cn/ (accessed on 1 November 2023). The climate production potential (CPP) was calculated using the Tharnthwaite Memorial model in OpenGMS https://geomodeling.njnu.edu.cn/ (accessed on 28 January 2024). The potential evapotranspiration (PET) data were obtained from a previous publication [32].

#### 2.3.5. Relief Covariates

The digital elevation model (DEM) data were derived from the Geospatial Data Cloud http://www.gscloud.cn/ (accessed on 12 January 2024). Then, SAGA GIS 7.8.2 [33] was used to extract the slope (SLP), aspect (APT), plan curvature (PLC), profile curvature (PRC), topographic wetness index (TWI), topographic position index (TPI), terrain ruggedness index (TRI), multiresolution index of ridge top flatness (MRRTF), and multiresolution index of valley bottom flatness (MRVBF) from the DEM data.

#### 2.3.6. Human Activity Covariates

The nighttime light (NL), particulate matter (PM10 and PM2.5), and gross domestic product (GDP, in millions of dollars) data were sourced from the National Earth System Science Data Center https://www.geodata.cn/ (accessed on 11 January 2024). The population density (PD) data came from the National Tibetan Plateau Science Data Center https://data.tpdc.ac.cn/ (accessed on 11 January 2024). The land use (TDLY) data were obtained from the Third National Land Survey.

### 2.4. Modeling Methodology

#### 2.4.1. Recursive Feature Elimination

**Figure 2:**
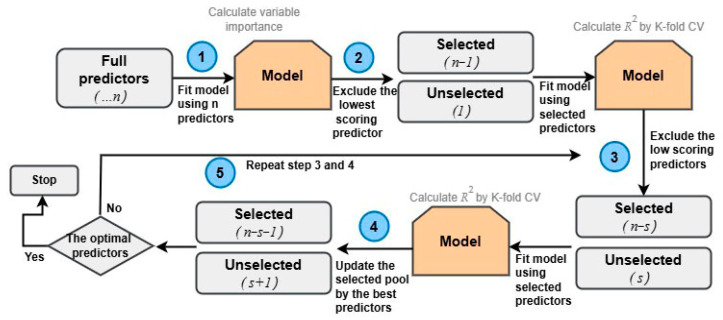
The diagram of recursive feature elimination.

The RFE algorithm is a common method tasked to select the most relevant predictors for machine learning models [34,35,36]. RFE works as follows: (1) input all the features into the model, and compute the model performance by a k-fold cross-validation and variable importance; (2) select the features with the lowest ranked performance metrics, and exclude them from the feature set; (3) train the model and calculate its performance metrics; and (4) repeat steps 2 and 3 until the number of features reaches a preset value or it is not possible to continue feature elimination (Figure 2). In this study, the RFE algorithm was implemented through the *RFE* package in the Python scikit-learn library [37], using RF as an internal model.

#### 2.4.2. Random Forest

**Figure 3:**
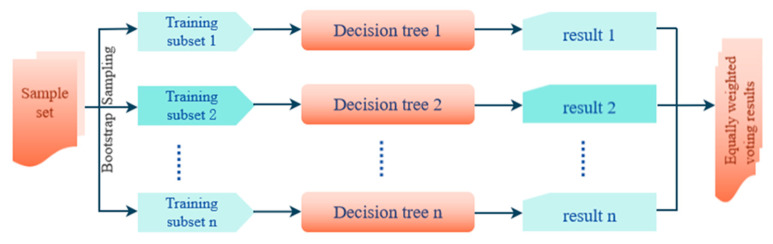
The framework of the random forest model.

The RF algorithm, initially developed by Breiman in 2001, is a supervised machine learning method with a wide application [38]. It uses bootstrap resampling to fit a large number of decision trees on the sub-samples of the training set, and applies weighted averaging to improve the accuracy of the prediction results (Figure 3). Compared to single models such as decision trees, the RF model is less sensitive to overfitting, multicollinearity, and missing or unbalanced data. The calculation formula used by the RF model is as follows:

(1) $$f\left(m_{t}\right)=\frac{1}{n}\sum_{i=1}^{n}T\left(m_{t}\right)$$

where $f\left(m_{t}\right)$ is the prediction result of the RF model; $m_{t}$ is the decision tree *t*; *n* is the number of decision trees; and $T\left(m_{t}\right)$ is the prediction result for the decision tree *t*. In this study, a Bayesian optimization algorithm was applied to tune the parameters of the RF model based on the criterion of the minimum root mean square error [39]. The RF model was executed using the *RandomForestRegressor* package in the Python scikit-learn library [40].

#### 2.4.3. Extreme Gradient Boosting

The XGBoost algorithm is a machine learning method implemented within the gradient boosting framework [41]. It continuously adds new decision trees to fit the residual of the previous decision tree and to predict the training sample *q*; finally, the corresponding results of all decision trees are added up to obtain the predicted value of sample *q*. Compared to the gradient boosting decision tree, XGBoost advances the loss function to an approximation of the second derivative and introduces a regularization term into the loss function. The calculation formula of the loss function is expressed as follows:

(2) $$L\left(\delta \right)=\sum_{i=1}^{n}l\left(y_{i}^{/},y_{i}\right)+\sum \Omega \left(f\right)$$

where *L(δ)* is the loss function; *n* is the number of samples; *l(*$y_{i}^{/}$*,*$y_{i}$*)* is the loss for a single sample, assuming it is a convex function; $y_{i}^{/}$ is the predicted value of the *i-*th sample; $y_{i}$ is the true value of the *i*-th sample; and $\sum \Omega \left(f\right)$ is the regularization term, defined as follows:

(3) $$\sum \Omega \left(f\right)=\gamma T+\frac{1}{2}\lambda {\left\|w\right\|}^{2}$$

where γ and λ are manually set parameters; w are the vectors formed by the values of all leaf nodes in the decision tree; and T is the number of leaf nodes. The calculation formula used by XGBoost is as follows:

(4) $$\hat{y}=\sum_{i=1}^{M}\lambda_{i}h_{i}(x)$$

where $\hat{y}$ is the prediction result of the XGBoost model; *M* is the total number of decision trees; $\lambda_{i}$ is the weight of the *i*-th decision tree, also known as the learning rate or scaling parameter; and $h_{i}\left(x\right)$ is the prediction result of the *i*-th decision tree for sample *x*. In this study, the XGBoost model was implemented using the *XGBRegressor* package in the Python xgboost library [42].

#### 2.4.4. Model Performance Evaluation

The dataset was randomly divided into training and validation sets based on 10-fold cross-validation [43]. Then, the Bayesian optimization algorithm was used for the parameter tuning and the optimal parameters were substituted into the model for 100 iterations. To evaluate the prediction performance of the machine learning models integrated with variable selection for soil properties, we calculated the mean absolute error (*MAE*), root mean square error (*RMSE*), and coefficient of determination (*R*^2^) between the measured and predicted values at the validation sample points. The calculation formulae of the three performance metrics are as follows:

(5) $$RMSE=\sqrt{\frac{\sum_{i=1}^{n}{\left[z\left(x_{i}\right)-Z\left(x_{i}\right)\right]}^{2}}{n}}$$

(6) $$MAE=\frac{1}{n}\sum_{i=1}^{n}\left|z(x_{i})-Z(x_{i})\right|$$

(7) R2=1−∑i=1nzxi−Zxi2∑i=1nZxi−Z−xi2

where n is the number of sample points in the validation set; $z\left(x_{i}\right)$ is the predicted value at the sample point *i*; $Z\left(x_{i}\right)$ is the measured value at the sample point *i*; and Z−xi is the average of the measured values at the sample points 1 to *i*. Smaller MAE and RMSE values, as well as *R*^2^ values closer to 1, indicate a higher prediction accuracy and consequently a superior model performance [44,45].

The global uncertainty in the models’ predictive ability was assessed using the method described by Zhou et al. [46]. Each prediction model was run 100 times. Then, the mean and standard deviation (SD) of the prediction results from the 100 runs were calculated based on pixels. The mean plot was used as the final prediction result, and the SD plot was used to assess the uncertainty in each prediction model.

## 3. Results

### 3.1. Descriptive Statistics of the Soil Properties

The descriptive statistics of the soil properties in the Haihun River sub-watershed are provided in Table 2. Across the sample points, the SOC content ranged from 0.10 to 2.82 g·kg^−1^ with a mean of 1.27 g·kg^−1^.The STP content varied between 161.00 and 1103.00 mg·kg^−1^ with a mean of 505.53 mg·kg^−1^. The SAP content fell into the range of 0.12–59.30 mg·kg^−1^ with a mean of 10.30 mg·kg^−1^. Based on their coefficients of variation, the SOC and STP contents showed moderate spatial variability, and the SAP content showed strong spatial variability. The data of all three soil properties basically obeyed normal distribution in terms of skewness and kurtosis (Supplementary Table S1–S3).

**Figure 4:**
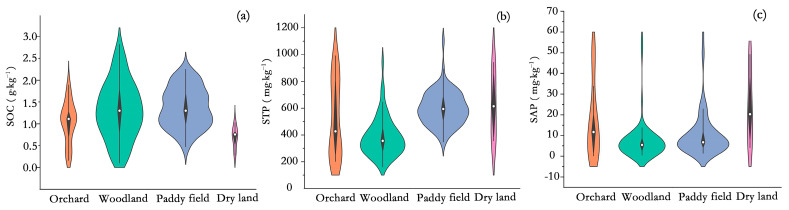
Distribution of soil organic carbon (SOC) (**a**), total phosphorus (STP) (**b**), and available phosphorus (SAP) (**c**) under different land use types.

The distribution of the soil properties under different types of land use is shown in Figure 4. There were minor differences in the distribution of the SOC content among various types of land use, with generally higher values in woodland, paddy fields, and orchards compared to dry land. The median of the STP content under different land use types was ranked in descending order, as follows: dry land > paddy field > orchard > woodland. The values of the SAP content were mainly distributed at low and medium levels, with a higher median in the dry land compared to other land use types.

### 3.2. Variable Selection

The optimal covariate combination for predicting the soil properties was determined based on the highest model performance (*R*^2^) of the RFE. Of the forty environmental covariates (Table 3), twelve covariates were retained in the RFE for the SOC (*R*^2^ = 0.86); these included three, one, two, one, two, and three covariates related to the soil, climate, organisms, relief, remote sensing, and human activity, respectively. As for the STP, the RFE (*R*^2^ = 0.89) retained eight covariates, with one, one, two, one, one, and two covariates related to the soil, climate, organisms, relief, remote sensing, and human activity, respectively. In the case of the SAP, the RFE (*R*^2^ = 0.91) only retained four covariates related to the relief (TPI), organisms (PET and NPP), and remote sensing (PCA1).

### 3.3. Model Accuracy

While the RFE determined the optimal combination of environmental predictors for each soil property, variable selection effectively reduced the model processing time. The performance of the RF and XGBoost models was indicated by the mean values of the *RMSE*, *MAE*, and *R*^2^ for 100 iterations based on a 10-fold cross-validation (Table 4). Generally, a low accuracy was observed for the models that contained all 40 environmental covariates (hereafter referred to as ‘all-variable models’). In particular, the XGBoost model for the SAP had the lowest accuracy, with an *RMSE* of 11.81 mg·kg^−1^, *MAE* of 9.02 mg·kg^−1^, and *R*^2^ of 0.15.

The RF and XGBoost models based on variable selection performed notably better than the all-variable models (Table 4). Specifically, the *R*^2^ values of the models for the SOC, STP, and SAP all substantially increased after variable selection. The *R*^2^ values of the RF and XGBoost models with variable selection reached 0.79 and 0.84 for the SOC, respectively; the corresponding *R*^2^ values were 0.70 and 0.77 for the SAP. Compared to the SOC and STP models, the STP models showed the greatest optimization and the highest accuracy after variable selection (RF_*R*^2^ = 0.86; XGBoost_*R*^2^ = 0.89). When the machine learning models were integrated with variable selection, the XGBoost model was improved in its prediction accuracy (*R*^2^) for the soil properties by 3.5%–10.0% compared to the RF model. Both of the two models showed an excellent prediction performance, with minor difference in their accuracy.

### 3.4. Spatial Distribution of the Soil Properties

**Figure 5:**
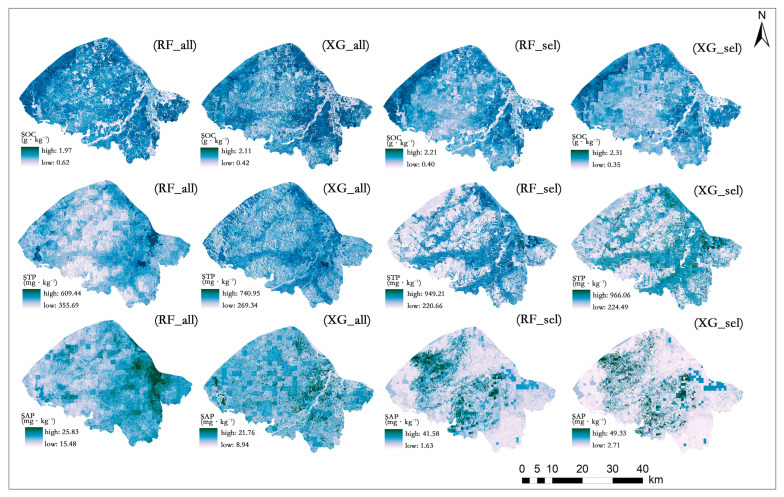
Spatial distribution of the soil properties predicted by different machine leaning models without (RF_all and XG_all) and with (RF_sel and XG_sel) variable selection.

The spatial distribution of the SOC, STP, and SAP in the study area was mapped based on the RF and XGBoost models without and with variable selection (Figure 5). No distinct spatial patterns were observed for any soil properties predicted by the all-variable models (RF_all and XG_all). The overall trends of the soil properties predicted by the different methods barely changed after variable selection. High values of the SOC were distributed in the northwestern and southeastern parts of the sub-watershed, with low values in the central part. The location of the high SOC areas was highly consistent with the distribution of water areas within the sub-watershed. The high SAP areas were concentrated in the central part of the sub-watershed, with a cloud-like distribution. All three soil properties showed a high spatial heterogeneity.

**Figure 6:**
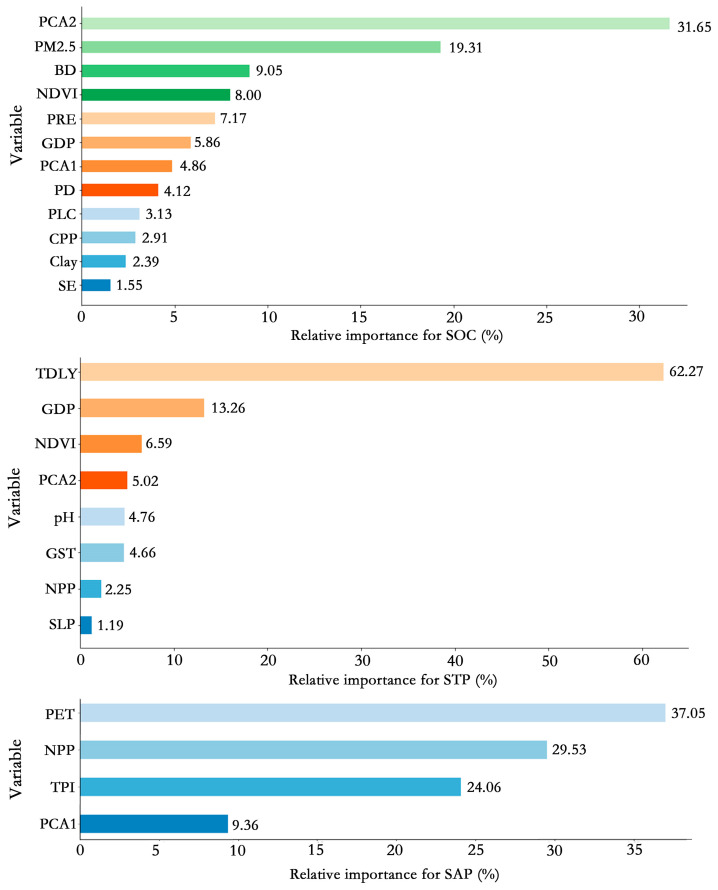
Relative importance of variables in the random forest model for soil properties. The variables are defined in Table 1.

The relative importance of the variables in the RF model was calculated. Among all types of variables, the significant variables for the SOC were mainly related to remote sensing (36.51%) and human activity (29.29%; Figure 6). PCA2 (31.65%) was the most important variable for the SOC, followed by PM2.5 (19.31%). The STP was prominently influenced by TDLY (62.27%) related to human activity. This affirms a high consistency between the spatial distributions of STP and the land use types in the study area. The organism covariates (66.58%) followed by the relief (24.06%) were important for the SAP, with PET accounting for the highest percentage (37.05%).

### 3.5. Model Uncertainty

**Figure 7:**
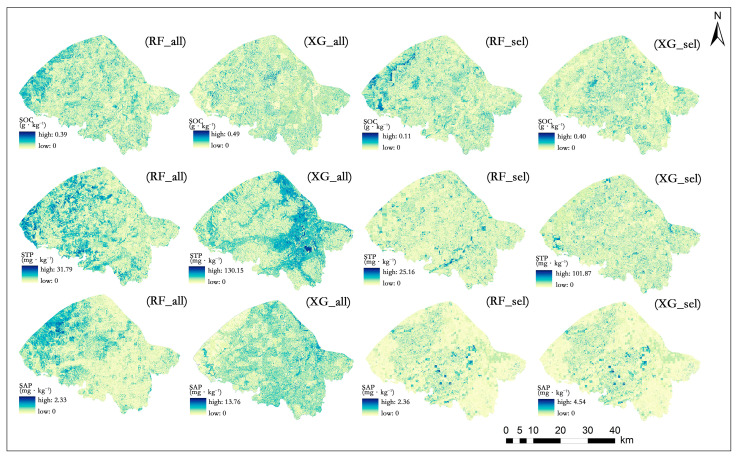
Spatial distribution of uncertainty in model predictions of soil properties.

The global uncertainty in model predictions was quantified using the SD of the predicted values for 100 iterations. The RF and XGBoost models with optimal variable selection (RF_sel and XG_sel) exhibited a lower uncertainty than the respective all-variable models (RF_all and XG_all; Figure 7). In the SOC prediction, the mean uncertainty estimates for the RF_sel model ranged from 0 to 0.11 g·kg^−1^, and the estimates for the XG_sel model were between 0 and 0.40 g·kg^−1^. In the STP prediction, the mean uncertainty estimates for the RF_sel and XG_sel models fell in the range of 0–25.16 mg·kg^−1^ and 0–101.87 mg·kg^−1^, respectively. In the SAP prediction, the range of mean uncertainty estimates was 0–2.36 mg·kg^−1^ for the RF_sel and 0–4.54 mg·kg^−1^ for the XG_sel model.

The results showed that all the variable selection models exhibited low levels of uncertainty. Despite their similar accuracy, the RF model had a lower uncertainty and was more robust than the XGBoost model to predict the soil properties. With regard to the spatial distribution pattern of uncertainty in the models’ predictions, relatively high estimates were found in the northwestern and east–central parts of the sub-watershed with fragmented land use types or large elevation changes (Figure 7).

## 4. Discussion

### 4.1. Benefits of Variable Selection

Before predicting the soil properties in the Haihun River sub-watershed, we selected the most relevant environmental covariates using RFE. The RFE algorithm eliminated 70%, 80%, and 90% of the initial covariates for the SOC, STP, and SAP, respectively. Despite no wide distinction in accuracy between the models, the model uncertainty decreased after variable selection (Figure 7). Our results show that eliminating the redundant variables prior to the modeling can improve the model parsimony and accuracy for digital soil mapping [47]. Therefore, the hypothesis proposed in this paper is validated.

Among the variables selected by RFE, frequent human activity (e.g., PM2.5) was likely to reduce the correlation between vegetation (e.g., NDVI) and the SOC. Particulate matter, such as PM2.5, could adhere to plant leaves, hindering leaf photosynthesis and inhibiting vegetation growth [48]. Notably, the number of relief covariates decreased after the RFE-based variable selection, because their data were extracted from the DEM with high multicollinearity. In contrast, multiple variables related to remote sensing, organisms, and human activity were retained, suggesting their possible roles in the prediction of soil properties at the regional scale.

### 4.2. Comparison of Model Performance

To predict multiple soil properties in the study area, we executed RF and XGBoost models using 40 environmental covariates and the optimal covariate combination. The RFE-optimized machine learning models exhibited significant superiority to the all-variable models in terms of prediction performance (Table 4). We additionally observed similar overall trends in the spatial distribution of each soil property predicted by the two models with variable selection (Figure 5). This consistency verifies the effectiveness and accuracy of the machine learning models developed in this study. The range of mean predicted values for the 100 iterations of the RF and XGBoost models was close to the statistical range of the soil properties in soil samples (Table 2), indicating that the selection of machine learning models was reliable. From a global distribution perspective, the prediction of soil properties by integrating machine learning and variable selection solves the problem of multicollinearity that may exist among variables in the all-variable prediction. From a local distribution perspective, the models developed in this study are able to capture rich, detailed information on multiple soil properties.

The XGBoost model with RFE-based variable selection showed a slightly higher accuracy than the RF model, despite no considerable difference between the two models. Specifically, in the SOC prediction, the RF model yielded a 13.02% higher RMSE and a 5.37% lower R^2^ than the XGBoost model, similar to the results of the STP and SAP predictions (Table 4). However, the RF model performed better than the XGBoost model with regard to the spatial distribution and uncertainty of the predicted soil properties. For example, the uncertainty estimates of the RF model were generally lower than those of the XGBoost model for the SOC prediction (Figure 7). Uncertainty mainly arises from heterogeneity in the spatial distribution of soil properties. Due to the highly variable environmental characteristics across regions, the correlations between soil properties and environmental covariates are complicated and unquantifiable. As a result, the lack of information for model fitness leads to a decrease in the model stability [49]. Additionally, biases in the resampling process may propagate into the prediction [50].

In summary, the RF model has the best prediction performance and can be considered as the optimal model for predicting the soil properties in the study area [51,52,53]. The maps of SOC, STP, and SAP based on the RF_sel model are provided as the final prediction results (Figure 5). This study presents useful information that could help select the method and optimize the accuracy of predicting the spatial distribution of soil properties at small scales.

### 4.3. Factors Controlling the Spatial Distribution of the Soil Properties

The spatial distribution patterns of soil properties can be explained by environmental covariates [54]. Based on the RF model, we identified distinct important environmental variables for the three soil properties in the study area. The important variables for the SOC content were mainly related to remote sensing and human activity. Among them, PCA2 had the strongest influence on the SOC content (Figure 6). PCA2 incorporates the spectral information (e.g., soil color) closely related to the SOC, and such information can effectively reflect the changes in the SOC content. Soil color, an indicator of soil health, is mainly determined by the SOC content [55]. Therefore, PCA2 can serve as a proxy for soil color and provide key information for the SOC modeling. The inclusion of this variable can improve the prediction accuracy and interpretability of the model. In the central part of the watershed, where the terrain is flat and agricultural land predominates, frequent cultivation activities tend to accelerate the decomposition of organic matter, leading to relatively lower levels of top soil organic carbon [56]. However, in the left region of the southeastern part, which is also predominantly arable land, the proximity to the river results in the accumulation of a significant amount of organic material eroded from upstream areas, thereby exhibiting a higher soil organic carbon content [57]. The northwestern part and the right region of the southeastern part, characterized by high vegetation cover and primarily forested, demonstrate elevated soil organic carbon levels due to substantial inputs of organic matter from the vegetation [58] (Figure 5).

We found that the STP content was predominantly controlled by land use related to human activity (Figure 6), with the STP and TDLY exhibiting consistent spatial patterns. Generally, land use type determines the input and output, as well as the cycling and transformation processes of the soil phosphorus [59]. For example, cropland is usually planted with crops that require large amounts of phosphorus fertilizer, leading to high levels of STP. In contrast, naturally vegetated areas (e.g., woodland and grassland) in the northwestern part of the study area may have low levels of STP due to phosphorus depletion and immobilization by the vegetation. A multitude of studies have shown that land use is a major factor driving the spatial variability of the soil phosphorus at local and regional scales [60,61], in support of our finding.

Organism covariates, particularly PET, had a prominent influence on the SAP content in the study area (Figure 6). PET describes the maximum evapotranspiration rate occurring under specific environmental conditions (e.g., temperature, humidity, solar radiation, and wind speed), when there is no limitation of water supply [62]. The phosphorus dynamics in the soil can be indirectly affected during evapotranspiration [63]. For example, strong evapotranspiration is likely to cause a decrease in the SM content, which in turn affects the dissolution and transport of phosphorus in the soil. Therefore, PET can be used as a predictor for the SAP.

Some relevant variables, such as SM, were not adopted in the RF model after variable selection by RFE, mainly due to the relatively low resolution of these data. Furthermore, the small watershed scale and flat topography limited the contribution of other environmental covariates, such as climate and relief, to the model.

### 4.4. Limitations and Deficiencies

The prediction accuracy of the machine learning models for the soil properties was affected by two major factors. First, the data of some environmental covariates were not highly refined, with raster data at a resolution of 1 km × 1 km. Due to the small scale of the study area, local spatial autocorrelation might be ignored at a low resolution, affecting the variable selection and reducing the prediction accuracy of the soil properties. Raster resampling for the purpose of unifying the scale of the environmental covariates also led to a reduction in data accuracy and information loss.

Second, insufficient environmental covariates were considered in this study. Bioclimatic covariates were not included as climate covariates. Temperature, which affects soil microbial decomposition processes, is more representative of the impacts of climate on the soil nutrients [64]. Additionally, there are various agricultural production activities in the study area, and the remote sensing covariates only represent agricultural activity to a certain extent. The inclusion of more agricultural activity factors (e.g., cropping system and fertilizer application patterns) as environmental covariates could improve the prediction accuracy and performance of the models.

## 5. Conclusions

In this study, we predicted the spatial distribution patterns of the soil organic carbon, total phosphorus, and available phosphorus contents in the Haihun River sub-watershed. To improve the prediction performance, we incorporated variable selection into two machine learning models. Recursive feature elimination was used to eliminate any redundant variables and to develop the optimal prediction models for multiple soil properties. The machine learning models with variable selection showed a notably improved performance compared to the all-variable models.

The spatial distribution of the soil properties exhibited consistent overall trends based on the random forest (RF) and extreme gradient boosting (XGBoost) models with variable selection. This consistency indicates the effectiveness and accuracy of the machine learning models used in this study. There was no distinct difference in the prediction accuracy of the soil properties between the two machine learning models. Nevertheless, the RF model presented a relatively low prediction uncertainty and a more robust predictive ability.

Among the variables included in the model predictions, remote sensing covariates (especially principal component 2) predominantly controlled the distribution of the soil organic carbon. Human activity covariates (mainly land use) played a major role in governing the distribution of soil total phosphorus. Organism covariates (represented by potential evapotranspiration) were the most important factors affecting the distribution of the soil available phosphorus. The results can provide guidance on soil management in the study area and serve as a methodological reference for the prediction of soil properties in other similar sub-watersheds.

## Figures and Tables

**Table 1:** List of environmental covariates in the database.

Type	Covariate	Abbreviation	Scale	Source
Soil (8)	Soil bulk density	BD	30 m	Calculated from sample data
	Soil pH	pH	30 m	Calculated from sample data
	Sand content	Sand	250 m	https://www.geodata.cn/ (accessed on 5 November 2023)
	Silt content	Silt	250 m	https://www.geodata.cn/ (accessed on 5 November 2023)
	Clay content	Clay	250 m	https://www.geodata.cn/ (accessed on 5 November 2023)
	Soil erosion	SE	30 m	[30]
	Soil moisture	SM	1000 m	https://data.tpdc.ac.cn/ (accessed on 12 January 2024)
	Bare soil index	BSI	30 m	Extracted from Landsat 8 data
Climate (7)	Maximum temperature	Tmax	30 m	[31]
	Mean temperature	Tmean	30 m	[31]
	Minimum temperature	Tmin	30 m	[31]
	Mean wind speed	WIN	1000 m	https://www.resdc.cn/ (accessed on 1 November 2023)
	Mean ground temperature	GST	1000 m	https://www.resdc.cn/ (accessed on 1 November 2023)
	Mean relative humidity	RHU	1000 m	https://www.resdc.cn/ (accessed on 1 November 2023)
	Precipitation	PRE	30 m	[31]
Remote sensing (3)	Principal component 1	PCA1	30 m	Extracted from Landsat 8 data
	Principal component 2	PCA1	30 m	Extracted from Landsat 8 data
	Principal component 3	PCA3	30 m	Extracted from Landsat 8 data
Organisms (6)	Normalized difference vegetation index	NDVI	30 m	https://www.nesdc.org.cn/ (accessed on 19 July 2023)
	Net primary productivity	NPP	500 m	https://www.geodata.cn/ (accessed on 1 November 2023)
	Net ecosystem productivity	NEP	1000 m	https://www.geodata.cn/ (accessed on 1 November 2023)
	Gross primary productivity	GPP	1000 m	https://www.geodata.cn/ (accessed on 1 November 2023)
	Climate production potential	CPP	30 m	https://geomodeling.njnu.edu.cn/ (accessed on 28 January 2024)
	Potential evapotranspiration	PET	30 m	[32]
Relief (10)	Elevation	DEM	30 m	http://www.gscloud.cn/ (accessed on 12 January 2024)
	Slope	SLP	30 m	Extracted from DEM data
	Aspect	APT	30 m	Extracted from DEM data
	Topographic wetness index	TWI	30 m	Extracted from DEM data
	Plan curvature	PLC	30 m	Extracted from DEM data
	Profile curvature	PRC	30 m	Extracted from DEM data
	Topographic position index	TPI	30 m	Extracted from DEM data
	Topographic ruggedness index	TRI	30 m	Extracted from DEM data
	Multiresolution index of ridge top flatness	MRRTF	30 m	Extracted from DEM data
	Multiresolution index of valley bottom flatness	MRVBF	30 m	Extracted from DEM data
Human activity (6)	Land use	TDLY	30 m	Third National Land Survey
	Nighttime light	NL	500 m	https://www.geodata.cn/ (accessed on 11 January 2024)
	Particulate matter 10	PM10	1000 m	https://www.geodata.cn/ (accessed on 11 January 2024)
	Particulate matter 2.5	PM2.5	1000 m	https://www.geodata.cn/ (accessed on 11 January 2024)
	Population density	PD	1000 m	https://data.tpdc.ac.cn/ (accessed on 11 January 2024)
	Gross domestic product	GDP	km	https://www.geodata.cn/ (accessed on 11 January 2024)

**Table 2:** Descriptive statistics of the soil properties at the sample points.

Soil Property *	Min	Max	Mean	Standard Deviation	Coefficient of Variation (%)	Skewness	Kurtosis
SOC (g·kg^−1^)	0.10	2.82	1.27	0.56	44.09	0.16	2.64
STP (mg·kg^−1^)	161.00	1103.00	505.53	192.17	38.01	0.42	2.73
SAP (mg·kg^−1^)	0.12	59.30	10.30	11.95	116.02	0.32	3.84

* SOC, soil organic carbon; STP, soil total phosphorus; and SAP, soil available phosphorus.

**Table 3:** Model performance of recursive feature elimination for soil properties.

Soil Property	Variables before Selection	Variables after Selection		*R* ^2^
	Number	Type	Number	
SOC	40	Soil	3	0.86
		Climate	1	
		Organisms	2	
		Relief	1	
		Remote sensing	2	
		Human activity	3	
STP	40	Soil	1	0.89
		Climate	1	
		Organisms	2	
		Relief	1	
		Remote sensing	1	
		Human activity	2	
SAP	40	Organisms	2	0.91
		Relief	1	
		Remote sensing	1	

**Table 4:** Model prediction accuracy for the soil properties in the study area.

Soil Property	Model *	*RMSE*	*MAE*	*R* ^2^
SOC	RF_all	0.41	0.33	0.45
	RF_sel	0.25	0.17	0.79
	XG_all	0.44	0.36	0.37
	XG_sel	0.22	0.12	0.84
STP	RF_all	171.91	136.82	0.20
	RF_sel	71.21	50.01	0.86
	XG_all	163.76	128.10	0.27
	XG_sel	62.67	37.86	0.89
SAP	RF_all	14.22	12.85	0.27
	RF_sel	6.54	3.94	0.70
	XG_all	11.81	9.02	0.15
	XG_sel	5.69	3.05	0.77

* RF_all and RF_sel represent the random forest models with all variables and selected variables, respectively; and XG_all and XG_sel represent the extreme gradient boosting models with all variables and selected variables, respectively. The models’ prediction accuracy was evaluated in terms of the mean absolute error (*MAE*), root mean square error (*RMSE*), and coefficient of determination (*R*^2^).

## Data Availability

The data are available from the corresponding author upon reasonable request.

## Supplementary Materials

The following supporting information can be downloaded at: https://www.mdpi.com/article/10.3390/s24123784/s1, Table S1: Multicollinearity diagnostic of SOC; Table S2: Multicollinearity diagnostic of STP; Table S3: Multicollinearity diagnostic of SAP.
