# Supplementary material for: Mapping Soil Properties in the Haihun River Sub-Watershed, Yangtze River Basin, China, by Integrating Machine Learning and Variable Selection
Source: Sensors (Basel). 2024 Jun 11;24(12):3784. doi: 10.3390/s24123784 (PMC11207289; doi:10.3390/s24123784)
Supplement: Supplementary file 1 [file sensors-24-03784-s001.zip › sensors-2979191-supplementary.pdf]

1.Multicollinearity diagnostic of SOC

Ratio[a]									
		Unstandardised coefficient		Standardised coefficient			Statistic of covariance	
Model		B	Standard Error	Beta	t	Significance	Tolerance	VIF
1	(Constant)	-117.212	118.197		-.992	.323		
	APT	.000	.000	-.078	-1.075	.284	.738	1.354
	DEM	.000	.006	.010	.054	.957	.106	9.434
	MRRTF	-.013	.032	-.039	-.415	.679	.443	2.258
	MRVBF	.045	.024	.224	1.902	.059	.281	3.559
	PLC	.473	.150	.266	3.154	.002	.546	1.830
	PRC	.282	.172	.165	1.641	.103	.385	2.601
	SLP	-.003	.023	-.014	-.123	.902	.317	3.159
	TRI	.054	.047	.111	1.133	.259	.407	2.460
	TPI	.002	.083	.003	.024	.981	.298	3.355
	TWI	-1.819E-5	.000	-.092	-1.224	.223	.691	1.448
	GST	1.053	1.569	.321	.671	.503	.017	58.880
	PRE	.004	.002	.455	2.232	.027	.093	10.714
	RHU	.129	.883	.100	.146	.884	.008	120.039
	Tmax	1.598	1.290	.304	1.238	.218	.064	15.523
	Tmean	-1.434	1.311	-.279	-1.094	.276	.060	16.691
	Tmin	.581	1.191	.138	.488	.627	.049	20.465
	WIN	3.038	4.025	.249	.755	.452	.036	28.020
	GDP	-3.178E-6	.000	-.001	-.007	.994	.226	4.415
	PD	.000	.000	-.160	-1.715	.089	.444	2.251
	PM 10	-1.137	1.955	-.490	-.581	.562	.005	182.517
	PM 2.5	2.581	4.063	.602	.635	.526	.004	231.282
	NL	-.035	.057	-.094	-.622	.535	.170	5.874
	CPP	.017	.022	.534	.764	.446	.008	125.741
	GPP	8.188	3.878	.936	2.111	.037	.020	50.549
	NDVI	.007	.002	.321	3.431	.001	.445	2.249
	NPP	-1.865E-5	.000	-.107	-1.441	.152	.703	1.423
	PET	.001	.011	.017	.092	.927	.110	9.080
	BD	-.874	.240	-.264	-3.639	.000	.740	1.351
	Clay	-.001	.002	-2.433	-.664	.508	.000	3455.659
	pH	-.044	.112	-.038	-.396	.692	.425	2.351
	Sand	.001	.002	2.026	.668	.505	.000	2365.644

	SE	.000	.000	.150	1.773	.079	.543	1.843
	Silt	.000	.001	.344	.140	.889	.001	1549.094
	SM	-28.876	18.128	-.259	-1.593	.114	.147	6.785
	PCA1	-.002	.002	-.155	-1.361	.176	.300	3.336
	PCA2	.001	.001	.128	1.066	.288	.268	3.736
	PCA3	.000	.002	-.024	-.160	.874	.171	5.861
	TDLY	.012	.015	.065	.772	.441	.541	1.848
	BSI	-.001	.003	-.043	-.285	.776	.171	5.844

a. Implicit variable：SOC

2.Multicollinearity diagnostic of STP

Ratio[a]

Model		Unstandardised coefficient		Standardised coefficient			Statistic of covariance	
		B	Standard Error	Beta	t	Significance	Tolerance	VIF
1	(Constant)	-17402.954	40641.124		-.428	.669		
	BSI	-1.227	1.100	-.171	-1.115	.267	.160	6.241
	PCA1	.416	.519	.088	.801	.425	.315	3.179
	PCA3	.017	.844	.003	.020	.984	.158	6.342
	PCA2	.257	.342	.084	.753	.453	.299	3.343
	APT	-.152	.137	-.079	-1.106	.271	.737	1.357
	DEM	-4.372	2.348	-.394	-1.862	.065	.084	11.887
	MRRTF	-26.176	10.998	-.223	-2.380	.019	.430	2.326
	MRVBF	15.231	7.971	.220	1.911	.058	.284	3.519
	PLC	-68.600	51.415	-.112	-1.334	.184	.534	1.872
	PRC	-38.368	58.107	-.065	-.660	.510	.382	2.616
	SLP	4.354	7.893	.061	.552	.582	.312	3.205
	TRI	-13.955	16.147	-.083	-.864	.389	.410	2.436
	TPI	36.214	29.378	.144	1.233	.220	.277	3.614
	TWI	-.004	.005	-.058	-.799	.426	.702	1.424
	GST	-416.679	611.684	-.326	-.681	.497	.016	61.092
	PRE	1.376	.620	.453	2.217	.028	.090	11.122
	RHU	-61.948	297.434	-.137	-.208	.835	.009	114.271
	Tmax	68.857	450.134	.038	.153	.879	.060	16.731
	Tmean	377.570	441.277	.220	.856	.394	.057	17.544
	Tmin	-216.663	419.238	-.150	-.517	.606	.044	22.535
	WIN	678.012	1357.304	.157	.500	.618	.038	26.351
	GDP	-.202	.112	-.144	-1.799	.074	.585	1.711

	PD	.002	.037	.005	.045	.964	.294	3.406
	PM10	-201.241	665.047	-.246	-.303	.763	.006	176.223
	PM2.5	296.043	1386.196	.197	.214	.831	.004	227.007
	NL	.019	.020	.076	.955	.341	.587	1.704
	CPP	5.873	7.777	.535	.755	.451	.007	133.645
	GPP	1290.871	1348.015	.426	.958	.340	.019	52.625
	NDVI	.824	.693	.109	1.189	.237	.448	2.232
	NPP	-.004	.004	-.065	-.889	.375	.707	1.413
	PET	-.890	4.066	-.043	-.219	.827	.096	10.464
	BD	-131.370	80.365	-.118	-1.635	.104	.719	1.391
	Clay	.476	.648	2.721	.735	.464	.000	3648.114
	pH	94.142	36.706	.225	2.565	.011	.490	2.040
	Sand	-.076	.531	-.437	-.144	.886	.000	2464.492
	SE	-.044	.033	-.120	-1.329	.186	.463	2.162
	Silt	-.357	.419	-2.069	-.851	.396	.001	1572.261
	SM	5698.889	5785.649	.151	.985	.326	.161	6.213
	TDLY	-5.381	5.057	-.088	-1.064	.289	.554	1.805

a. Implicit variable：STP

3.Multicollinearity diagnostic of SAP

Ratio[a]

		Unstandardised coefficient		Standardised coefficient			Statistic of covariance	
Model		B	Standard Error	Beta	t	Significance	Tolerance	VIF
1	(Constant)	-3161.959	2896.892		-1.092	.277		
	APT	.008	.010	.068	.814	.417	.718	1.392
	DEM	.162	.171	.234	.948	.345	.082	12.195
	MRRTF	-2.277	.802	-.308	-2.839	.005	.424	2.358
	MRVBF	1.219	.579	.283	2.106	.037	.276	3.618
	PLC	-.841	3.632	-.022	-.232	.817	.546	1.830
	PRC	1.460	4.203	.039	.347	.729	.393	2.546
	SLP	-.976	.559	-.219	-1.747	.083	.316	3.163
	TRI	2.311	1.154	.217	2.003	.047	.423	2.365
	TPI	-.202	2.220	-.012	-.091	.928	.269	3.722
	TWI	-.001	.000	-.205	-2.493	.014	.737	1.356
	GST	18.134	44.037	.230	.412	.681	.016	62.633
	PRE	-.009	.044	-.047	-.197	.844	.086	11.623

RHU	19.152	21.209	.690	.903	.368	.009	117.140
Tmax	34.249	31.936	.314	1.072	.285	.058	17.175
Tmean	20.874	31.967	.198	.653	.515	.054	18.552
Tmin	38.006	29.823	.432	1.274	.205	.043	23.041
WIN	78.877	96.410	.295	.818	.415	.038	26.058
GDP	.001	.007	.016	.167	.868	.509	1.966
PD	.001	.003	.072	.550	.583	.292	3.420
PM10	40.163	49.480	.809	.812	.418	.005	199.539
PM2.5	-82.234	101.800	-.904	-.808	.421	.004	251.345
NL	.000	.001	.028	.308	.759	.592	1.690
CPP	-.587	.569	-.875	-1.031	.304	.007	144.576
GPP	-129.507	97.787	-.701	-1.324	.188	.018	56.260
NDVI	-.040	.050	-.086	-.810	.420	.442	2.261
NPP	-1.352E-5	.000	-.004	-.043	.966	.714	1.400
PET	.104	.273	.082	.380	.705	.106	9.469
BD	1.912	5.638	.028	.339	.735	.751	1.331
Clay	.024	.045	2.653	.523	.602	.000	5161.653
pH	2.792	2.609	.114	1.070	.286	.435	2.299
Sand	.009	.038	1.026	.243	.809	.000	3594.816
SE	.001	.002	.027	.262	.794	.477	2.096
Silt	-.032	.030	-3.598	-1.053	.294	.000	2346.861
SM	194.439	407.744	.083	.477	.634	.163	6.143
BSI	-.123	.079	-.282	-1.560	.121	.152	6.561
PCA1	.039	.037	.131	1.049	.296	.318	3.145
PCA2	.013	.024	.071	.542	.589	.292	3.425
PCA3	-.143	.061	-.419	-2.337	.021	.155	6.453
TDLY	.488	.367	.126	1.329	.186	.555	1.802

a. Implicit variable：SAP
